# The multiple sclerosis prodrome is just unspecific symptoms in radiologically isolated syndrome patients – No

**DOI:** 10.1177/13524585211035951

**Published:** 2021-09-08

**Authors:** Helen Tremlett, Darin T Okuda, Christine Lebrun-Frenay

**Affiliations:** Division of Neurology, Faculty of Medicine and the Djavad Mowafaghian Centre for Brain Health, University of British Columbia, UBC Hospital, Vancouver, BC, Canada; Department of Neurology, UT Southwestern Medical Center, Dallas, TX, USA; CRCSEP, UR2CA-URRIS Neurology, CHU Pasteur 2, Nice Cote d’Azur University, Nice, France

‘That which can be asserted without evidence, can be dismissed without evidence’.

**– Christopher Hitchens**

Do we have sufficient evidence to dismiss the multiple sclerosis (MS) prodrome as unspecific (vague) symptoms in a person with the radiologically isolated syndrome (RIS)? Unfortunately, the short answer is ‘NO’.

The Oxford Dictionary defines a prodrome as ‘an early symptom indicating the onset of a disease or illness’. Prodromal phases are well recognized for other neurological diseases, with criteria, including clinical markers, developed to identify individuals in the prodromal phase.^1,2^ The definition of a prodromal phase may also encompass a period of disturbance, representing a deviation from a person’s previous state of health that eventually leads to the onset of the disease. In contrast, RIS, formally named in 2009, is defined by the absence of symptoms – either neurological or suggestive of inflammatory demyelinating disease, as well as the presence of magnetic resonance imaging (MRI) findings corresponding to the 2005 dissemination in space criteria.^3^ Thus, while formal criteria to define and diagnose RIS exist, there are no such standards for prodromal MS.^4^ Furthermore, until recently, neither RIS nor a prodromal phase for MS was even thought to exist.^3–5^

RIS is typically identified after an MRI is performed for a reason other than for exploring typical MS symptoms associated with central nervous system (CNS) demyelinating disease.^3^ Symptoms reported at the first RIS-defining MRI have included migraine/headache, musculoskeletal pain and mood fluctuations.^3,6^ Comprehensive historical data from individuals with RIS are closely evaluated to ensure the absence of clinical symptomatology suggestive of inflammatory demyelinating events. The proportion of individuals that fall in these symptom categories and subsequent risk of evolution to MS remains unknown. Nonetheless, risk factors for clinical conversion have been identified, and, based on current evidence, the motive for the index MRI was not one of them.^6^ However, the inclusion of a much larger number of study subjects with reasons for MRI that overlap with those identified within the MS prodrome may prove otherwise. Based on a 2020 study, approximately half (51%) of the 451 RIS participants referred to a centre specialized in MS care in the United States or Europe developed MS after a median of 10 years.^6^ Younger age, a cerebrospinal fluid (CSF) profile suggestive of CNS demyelination, the presence of spinal cord or infratentorial lesions on the index scan were independent risk factors for clinical conversion, with an 87% risk of conversion for individuals having all factors.^6^

So where does RIS fit with the MS prodrome? In short, we do not currently know. We do know that formal recognition of RIS^3^ predated the recent interest in the MS prodrome.^4,7^ Both RIS and prodromal MS are likely reflective of deficiencies in our current diagnostic criteria for, and recognition of, MS. In the general population, we know that individuals who developed MS had an increase in healthcare use in the years leading up to MS onset, suggestive of a prodromal phase.^7^ Reasons for these increases included mental health issues, skin-related disorders, sleep alterations, sensory disturbances and bladder dysfunction.^8,9^ In these studies, while MRI-related findings for participants were not available, it would be reasonable to hypothesize that many could also have demyelinating lesions in the CNS. Studies based on healthcare utilization also suggest that misdiagnoses and missed opportunities for earlier recognition of MS are apparent, as well as non-neurological and atypical symptoms pre-dating classical MS onset.^8–10^ Thus, an improved understanding of all these features offers another path towards understanding the early events leading up to the onset and recognition of MS, and will help to optimize diagnostic approaches and treatment strategies.

The recognition of RIS is typically a chance finding on an MRI scan, such as after a cranial trauma or a vehicle accident. Whether a prodromal phase pre-emptive of MS exists in individuals with RIS is currently unknown. This is not surprising, given how nascent our understanding is of the MS prodrome and RIS. Furthermore, it is unknown whether RIS and the MS prodrome are on a continuum or represent entirely distinct entities. For example, RIS subjects who report heat intolerance, mood disorders, headache or paroxysmal symptoms, yet present with a normal neurological examination, could already be in the prodromal phase of MS. However, these are relatively non-specific issues, which are also common in the general population without MS.^8^ Thus, unsurprisingly, the presence of one of these non-specific features, such as headache or migraine, was not found to be valuable predictors of conversion from RIS to MS.^6^ Likely, a combination of features and factors will be needed. These could range from clinical signs and symptoms, demographics (e.g. sex and age), relevant serum biomarker(s) and/or a family history or genetic risk score. A similar approach is used in Parkinson’s disease, whereby the probability that an individual has prodromal Parkinson’s disease can be estimated (for research purposes) using validated criteria.^2^ Such research criteria, if developed for MS, may also benefit from including the presence of RIS. The study of the prevalence of MRI features supportive of radiological criteria for MS within the MS prodromal phase is also needed. In addition, there is a need for significantly larger numbers of subjects with RIS, with a range of MRI indications, to understand if symptoms that may qualify as being prodromal impact risk for clinical evolution to MS. Worldwide collaboration is needed. Combined, all these efforts may lead to the development of appropriate tools to facilitate in the detection, earlier recognition and prompt management of MS.

## Conclusion

Formal recognition of RIS predates the recent interest in the MS prodrome.^3,4^ Thus, it comes as no surprise that little is known of how RIS might overlap with the MS prodrome. So, do we have sufficient evidence to dismiss the notion that ‘the MS prodrome is just unspecific symptoms in RIS patients’? The answer is a resounding ‘NO’. A dismissive approach without rigorous study is a disservice to our current and future patients. A combined effort is needed. Learning from both RIS and the emerging field of prodromal MS provides opportunity for earlier MS detection and management and possibly even prevention of MS in the future.^4^
